# Fermentation Conditions that Affect Clavulanic Acid Production in *Streptomyces clavuligerus*: A Systematic Review

**DOI:** 10.3389/fmicb.2016.00522

**Published:** 2016-04-22

**Authors:** Hooi-Leng Ser, Jodi Woan-Fei Law, Nathorn Chaiyakunapruk, Sabrina Anne Jacob, Uma Devi Palanisamy, Kok-Gan Chan, Bey-Hing Goh, Learn-Han Lee

**Affiliations:** ^1^School of Pharmacy, Monash University MalaysiaBandar Sunway, Malaysia; ^2^Biomedical Research Laboratory, Jeffrey Cheah School of Medicine and Health Sciences, Monash University MalaysiaBandar Sunway, Malaysia; ^3^Department of Pharmacy Practice, Faculty of Pharmaceutical Sciences, Center of Pharmaceutical Outcomes Research, Naresuan UniversityPhitsanulok, Thailand; ^4^School of Pharmacy, University of Wisconsin–MadisonMadison, WI, USA; ^5^School of Population Health, University of QueenslandBrisbane, QLD, Australia; ^6^Division of Genetics and Molecular Biology, Faculty of Science, Institute of Biological Sciences, University of MalayaKuala Lumpur, Malaysia; ^7^Center of Health Outcomes Research and Therapeutic Safety (Cohorts), School of Pharmaceutical Sciences, University of PhayaoPhayao, Thailand

**Keywords:** clavulanic acid, clavulanate, *Streptomyces clavuligerus*, fermentation, systematic review

## Abstract

The β-lactamase inhibitor, clavulanic acid is frequently used in combination with β-lactam antibiotics to treat a wide spectrum of infectious diseases. Clavulanic acid prevents drug resistance by pathogens against these β-lactam antibiotics by preventing the degradation of the β-lactam ring, thus ensuring eradication of these harmful microorganisms from the host. This systematic review provides an overview on the fermentation conditions that affect the production of clavulanic acid in the firstly described producer, *Streptomyces clavuligerus*. A thorough search was conducted using predefined terms in several electronic databases (PubMed, Medline, ScienceDirect, EBSCO), from database inception to June 30th 2015. Studies must involve wild-type *Streptomyces clavuligerus*, and full texts needed to be available. A total of 29 eligible articles were identified. Based on the literature, several factors were identified that could affect the production of clavulanic acid in *S. clavuligerus*. The addition of glycerol or other vegetable oils (e.g., olive oil, corn oil) could potentially affect clavulanic acid production. Furthermore, some amino acids such as arginine and ornithine, could serve as potential precursors to increase clavulanic acid yield. The comparison of different fermentation systems revealed that fed-batch fermentation yields higher amounts of clavulanic acid as compared to batch fermentation, probably due to the maintenance of substrates and constant monitoring of certain entities (such as pH, oxygen availability, etc.). Overall, these findings provide vital knowledge and insight that could assist media optimization and fermentation design for clavulanic acid production in *S. clavuligerus*.

## Introduction

Microorganisms serve as attractive resources, owing to their ability to synthesize structurally-diverse substances with various bioactivities (Demain, 1999; Newman et al., 2000; Bérdy, 2005; Demain and Sanchez, 2009). These microbial natural products may be used as effective drug(s) or act as drug lead compounds that could be further modified and developed for higher efficacy. Within the Bacteria domain, actinomycetes showed unprecedented ability to produce potentially novel, clinically useful, secondary metabolites such as anticancer, antioxidants, antivirals and antibacterials (Ara et al., 2014; Lee et al., 2014a,b; Manivasagan et al., 2014; Azman et al., 2015; Ser et al., 2015a,b; Tan et al., 2015). These filamentous bacteria produce around 8700 antibiotics, with the majority of them derived from members of the *Streptomyces* genus (Bérdy, 2005; Demain and Sanchez, 2009; de Lima Procópio et al., 2012). As the largest antibiotic-producing genus, *Streptomyces* species are capable of producing different classes of antibiotics including aminoglycosides (e.g., streptomycin by *S. griseus*), macrolides (e.g., tylosin from *S. fradiae*), and β-lactams (e.g., cephamycin and clavulanic acid by *S. clavuligerus*) (Waksman et al., 1944; Brown et al., 1976; Reading and Cole, 1977; Okamoto et al., 1982).

The β-lactam antibiotics are one of the most popular classes of antibacterial agents, whose mechanism of action is via inhibition of bacterial cell wall synthesis (Page, 2012). Soon after the utilization of β-lactam antibiotics, a number of bacteria have been found to exhibit resistance to this class of drugs. One of the strategies deployed by this group of bacteria to survive against β-lactam antibiotics is by the production of a β-lactam-hydrolyzing enzyme – β-lactamase; which functions to neutralize these antibiotics by cleaving the β-lactam ring (Wilke et al., 2005; Toussaint and Gallagher, 2015). Thus, to overcome this resistance, β-lactamase inhibitors are often used in conjunction with β-lactam antibiotics as these compounds prevent the degradation of these antibiotics and increase the efficacy of these drugs (Saudagar et al., 2008).

Clavulanic acid was first purified as a novel β-lactamase inhibitor from *S. clavuligerus* ATCC 27064, which was isolated from South American soil in 1971 (Higgens and Kastner, 1971; Brown et al., 1976). This compound presents with a nucleus similar to that of penicillin, with notable differences such as lacking anacylamino side chain, containing oxygen in place of sulfur, and having a β-hydroxyethylidine substituent in the oxazolidine ring (Brown et al., 1976; Saudagar et al., 2008). Clavulanic acid or clavulanate, is commercially used along with amoxicillin (Augmentin) and this combination has been listed as an important antibacterial agents in the WHO list of essential medicines (2015) (Toussaint and Gallagher, 2015). This compound was first recovered from the fermentation process, which remains as one of the most frequently used strategies to manufacture important drugs and their intermediates for medicinal use. In order to facilitate the higher production of valuable compound as such, advanced fermentation technologies were subsequently developed, which included fed-batch fermentation systems (Thiry and Cingolani, 2002; Schmidt, 2005).

**Figure 1:**
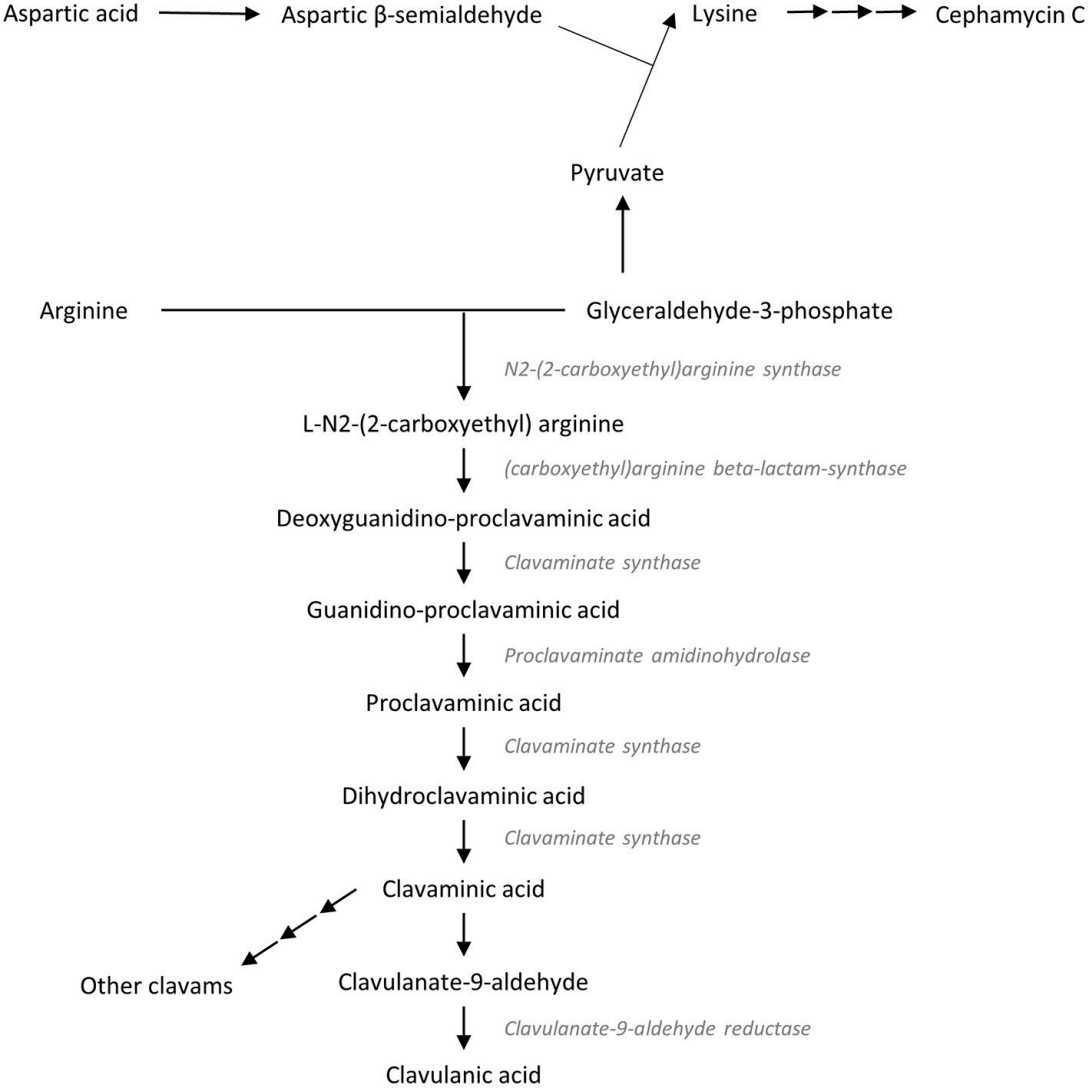
**Biosynthesis pathways of clavulanic acid (Kanehisa and Goto, 2000; Lynch and Yang, 2004; Kanehisa et al., 2016)**.

At the same time, researchers began to look into the biosynthesis pathway of clavulanic acid in an attempt to maximize its production (Figure 1). These efforts then resulted in the identification of two important precursors for clavulanic acid—arginine (C5 precursor) and glutaraldehyde-3-phosphate (C3 precursor) (Romero et al., 1986; Kanehisa and Goto, 2000; Kanehisa et al., 2016). Apart from clavulanic acid, *S. clavuligerus* is known to produce other clavams and cephamycin; as illustrated in Figure 1. As *S. clavuligerus* is unable to assimilate glucose, various compounds have been studied as C3 precursor candidates to ensure proper formulation of fermentation media and improve the yield of clavulanic acid (Aharonowitz and Demain, 1978; Garcia-Dominguez et al., 1989; Pérez-Redondo et al., 2010). Thus, this systematic review examined the effect of different fermentation conditions on the production of clavulanic acid in *S. clavuligerus*. Based on the available literature, our objective was to describe how additional supplements in basal medium, pH, as well as temperature affect the production of the β-lactamase inhibitor in *S. clavuligerus*, which in turn could assist and improve the development of fermentation media and/or systems for the production of this valuable antibiotic, clavulanic acid.

## Methods

This systematic review was carried out in accordance with the preferred reporting items for systematic reviews and meta-analyses (PRISMA) guidelines (Moher et al., 2009).

### Database search

Systematic searches were performed in the following databases: PubMed, Medline, ScienceDirect, and EBSCO. MeSH terms were “*Streptomyces*,” “*clavuligerus*” combined with “clavulanic acid” or “clavulanate.” We included studies from database inception to June 30th, 2015.

### Study selection and data extraction

Two reviewers (H-LS and JW-FL) independently screened and evaluated all titles and abstracts retrieved from the comprehensive search, based on the inclusion and exclusion criteria. The bibliographies of relevant studies were checked for additional publications. Full text of selected original articles were then obtained and reviewed. Any disagreements between the two reviewers were resolved by consensus. Studies providing data of clavulanic acid production in *Streptomyces clavuligerus* were included. Other inclusion criteria were: (1) studies must involve wild-type *Streptomyces clavuligerus*; (2) studies must describe fermentation conditions using batch and/or fed-batch fermentation strategies; and (3) studies must report the specific amount of clavulanic acid produced by *Streptomyces clavuligerus*. Studies conducted using *S. clavuligerus* mutants, studies conducted in organisms other than *S. clavuligerus*, and studies reporting only specific production of clavulanic acid; were excluded. We also excluded solid phase extraction studies, immobilization studies, and all reviews, conference abstracts, systematic reviews, meta-analyses, comments, and letters to the editor. The following information was extracted independently by the two reviewers from each study (Table 1): (1) study and year of publication, (2) fermentation type and volume, (3) oxygen control and/or airflow control, (4) fermentation/production media composition and pH, (5) addition of supplements (e.g., glycerol, starch, and vegetable oils), and (6) maximum clavulanic acid produced. Any discrepancies were discussed between both authors.

**Table 1:** **Effect of fermentation conditions on clavulanic acid production in *Streptomyces clavuligerus* (Max CA, maximum clavulanic acid; NBD, neutralized, bleached and deodorized; NB, neutralized and bleached; TN, total nitrogen; N.A., not available)**.

**References**		**Strain**	**Basal medium**	**Fermentation type**	**Fermenter volume, mL**	**Fermentation volume, mL**	**O_2_ control**	**pH**	**Temperature, °C**		**Shaking/Stirrer speed, rpm**	**Supplement**	**Max CA concentration^#^, mg/L**
									**Fixed**	**Gradient**			
1	Romero et al., 1986	NRRL 3585	Glycerol, sucrose, proline, NaCl, K_2_HPO_4_, CaCl_2_, MnCl_2_.4H_2_O, FeCl_3_.6H_2_O, ZnCl_2_, MgSO_4_.7H_2_O	Batch	N.A.	N.A.	No	7	N.A.	N.A.	N.A.	Arginine Ornithine	16 μg/mg DW^*^ 9 μg/mg DW^*^
2	Lebrihi et al., 1987	N.A.	Glycerol, L-asparagine, MgSO4, K_2_HPO_4_, FeSO_4_.7H_2_O, MnCl_2_.4H_2_O, ZnSO_4_.7H_2_O, CaCl_2_	Batch	1000/3000	200/600	No	6.9	28	–	250	Phosphate (2 mM) Phosphate (75 mM)	90 3
3	Belmar-Beiny and Thomas, 1991	N.A.	Glycerol, malt extract, bacteriological peptone, polypropylene glycol (antifoam)	Batch	7000	5000	0.5 vvm	7	26	–	490 990 1300	– – –	175 180 250
4	Lee and Ho, 1996	NRRL 3585	Proline, glutamic acid, NaCl, K_2_HPO_4_, CaCl_2_, MnCl_2_.4H_2_O, FeCl_3_.6H_2_O, AnCl_2_, MgSO_4_.7H_2_O	Batch	N.A.	40	No	8.3	27	–	220	Glycerol	0
								8.3				Sucrose	3.63
								9.1				Crude palm oil	1.26
								8.6				NBD palm olein	0.8
								8.5				NBD palm stearin	0.4
								8.0				NB palm-kernel olein	1.89
								6.5				NB palm-kernel stearin	2.99
								6.4				Oleic acid	0
								7				Palmitic acid	0.64
								7.1				Stearic acid	1.68
								5.78				Lauric acid	0.08
5	Ives and Bushell, 1997	NRRL 3585	Glycerol, NH_4_Cl, K_2_HPO_4_,KH_2_PO_4_, MgSO_4_.7H_2_O, FeSO_4_.7H_2_O CuCl_2_, CoCl_2_, CaCl_2_.2H_2_O, ZnCl_2_, MnCl_2_, Na_2_MoO_4_	Batch	N.A.	1500	N.A.	N.A.	30	–	750	C-limited (glycerol, 15 g/L)	0
												N-limited (NH_4_Cl, 1.5 g/L)	34
												P-limited (No K_2_HPO_4_, replaced with MOPS at 21 g/L)	35
6	Kwon and Kim, 1998	ATCC 27064	Starch-asparagine medium	Batch	50	20	No	7	27	–	250	–	39.83-49.79
												Methyl viologen (added after 38 h)	9.96-25.89
												menadione (added after 38 h)	47.80-67.71
												plumbagin (added after 38 h)	55.76-79.66
												phenazine methosulfate (added after 38 h)	63.73-103.56
												H_2_O_2_ (added after 38 h)	51.78-69.71
7	Gouveia et al., 1999	NRRL 3585	Glycerol, sucrose, proline, glutamic acid	Batch	500	50	No	6.5	28	–	250	–	41
			Glycerol, sucrose, proline, arginine									–	83
			Glycerol, peptone, K_2_HPO_4_, soy meal									–	472
			Glycerol, peptone, K_2_HPO_4_, Samprosoy 90NB									–	920
8	Thakur et al., 1999	NRRL 3585	Yeast extract, malt extract, peptone, glycerol	Batch	250	27	No	7	30	–	200	–	80
			K medium									–	100
			Dextrin, soyabean meal, casein hydrolysate, FeSO_4_									–	90
			Glycerol, sucrose, proline, glutamic acid									–	40
9	Gouveia et al., 2001	N.A.	Glycerol, K_2_HPO_4_	Batch	500	55	No	6.5	28	–	250	Soybean protein extract	1120
												Corn steep liquor, soybean protein extract	490
												Yeast extract, soybean protein extract	700
												Bacteriological peptone, soybean protein extract	900
10	Chen et al., 2002	ATCC 27064	Soymeal extract, peptone, K_2_HPO_4_	Batch	N.A.	100	No	7	28	–	200	Glycerol (10 g/L)	11.36
												Glycerol (15 g/L)	11.79
												Glycerol (20 g/L)	10.93
												Glycerol (30 g/L)	4.61
												Glycerol (40 g/L)	2.89
				Fed-batch	N.A.	100	No	7	28	–	200	–	115
												Glycerol (added up to 84 h, every 12 h)	230
												Glycerol (added up to 108 h, every 12 h)	255
												Glycerol (added up to 132 h, every 12 h)	270
			Soymeal extract, peptone, K_2_HPO_4_	Batch	5000	3000	1 vvm	7	28	–	500	–	230
				Fed-batch	5000	2000	1 vvm	7	28	–	500	Glycerol (added up to 108 h, every hour)	280
11	Chen et al., 2003	ATCC 27064	Glycerol, soy meal extract, peptone, KH_2_PO_4_	Batch	500	110	No	7	N.A.	N.A.	N.A.	–	115
												Ornithine	200
												Arginine	100
												Ornithine and arginine	125
				Fed-batch	500	110	No	7	N.A.	N.A.	N.A.	–	100
												Ornithine (added every 12 h)	110
												Arginine (added every 12 h)	210
												Glycerol (added every 12 h)	300
												Glycerol and arginine (added every 12 h)	130
												Glycerol and ornithine (added every 12 h)	200
12	Lynch and Yang, 2004	N.A.	Glycerol, MgSO_._7H_2_O, K_2_HPO_4_, FeSO_4_.7H_2_O, MnCl_2_.4H_2_O, ZnSO_4_.H_2_O, CaCl_2_	Batch	2000	200	1 vvm	6.9	30	–	700	L-lysine (1 g/L)	0
												L-lysine (20 g/L)	27
												L-lysine (1 g/L), degraded clavulanic acid	42
13	Lin et al., 2005	ATCC 27064	Glycerol, soy meal extract, peptone, KH_2_PO_4_	Batch	500	110	No	7	28	–	200	–	90
					500 (11 mm baffle height)							–	130
					500 (16 mm baffle height)							–	180
14	Maranesi et al., 2005	N.A.	Dextrin, soybean flour, malt extract, FeSO_4_.7H_2_O, MOPS buffer	Batch	3000	500	No	7	28	–	250	–	200
			Glycerol bacto peptone, soybean flour, MOPS buffer									–	100
			Starch, soybean flour, soybean oil, malt extract, K_2_HPO_4_,MOPS buffer, FeSO_4_.7H_2_O, ZnSO_4_.7H_2_O									–	458
			Glycerol, soybean flour, Samprosoy 90NB, malt extract, K_2_HPO_4_,MOPS buffer, MnCl_2_.4H_2_0, FeSO_4_.7H_2_O, ZnSO_4_.7H_2_0, CaCl_2_									–	170
			Soybean flour, soybean oil, malt extract, K_2_HPO_4_,MOPS buffer, FeSO_4_.7H_2_O, ZnSO_4_.7H_2_O									–	478
			soybean flour, malt extract, K_2_HPO_4_,MOPS buffer, FeSO_4_.7H_2_O, ZnSO_4_.7H_2_O	Batch	3000	500	No	7	28	–	250	Soybean oil (16 g/L)	420
												Soybean oil (23 g/L)	753
												Soybean oil (30 g/L)	722
												Corn oil (23 g/L)	680
												Sunflower oil (23 g/L)	660
15	Neto et al., 2005	ATCC 27064	Glycerol, Samprosoy 90NB, malt extract, K_2_HPO_4_,MgSO_4_.7H_2_O, MgCl_2_.4H_2_O, FeSO_4_.7H_2_O, ZnSO_4_.7H_2_O	Batch	N.A.	4000	0.5 vvm	6.8	28	–	800	–	194
				Fed-batch	N.A.	2500	0.5 vvm	6.8	28	–	800	Same composition as media with lower glycerol concentration (10 g/L)	404
16	Rosa et al., 2005	ATCC 27064	Glycerol, samprosoy 90NB, malt extract, yeast extract, K_2_HPO_4_, MgSO_4_.7H_2_O, MnCl_2_.4H_2_O, FeSO_4_.7H_2_O, ZnSO4.7H2O	Batch	N.A.	4000	0%	6.8± 0.1	28	–	300	–	0
							12%				600	–	254
							21%				800	–	475
							28%				1000	–	614
							43%				800	–	482
							50%				250-800	–	191
17	Wang et al., 2005^$^	ATCC 27064	Soy flour, glycerol, ornithine, K_2_HPO_4_, FeSO_4_, MgSO_4_	Batch	300	30	No	N.A.	28	–	250	Varied concentration of each components	217–526
18	Bushell et al., 2006	NRRL 3585	Glycerol, NH_4_Cl, K_2_HPO_4_, MOPS, MgSO_4_.7H_2_O, FeSO_4_.7H_2_O, CuCl_2_, CoCl, CaCl_2_.2H_2_O, ZnCl_2_,MnCl_2_, Na_2_MoO_4_	Batch	2500	1000	2 vvm	6.8± 0.2	30	–	750	–	94
19	Teodoro et al., 2006	ATCC 27064	Glycerol, bacto peptone, malt extract, yeast extract, K_2_HPO_4_, MgCl_2_.4H_2_O, MnCl_2_.4H_2_O, FeSO_4_.7H_2_O, ZnSO_4_.7H_2_O, silicone antifoam	Batch	4000	4000	0.5 vvm	6.8± 0.1	28	–	800	Samprosoy 90NB (10 g/L)	135
												Samprosoy 90NB (20 g/L)	380
												Samprosoy 90NB (30 g/L)	290
20	Ortiz et al., 2007	ATCC 27064	Glycerol, soybean oil (SO), K_2_HPO_4_, MnCl_2_.4H_2_O, FeSO_4_.7H_2_O, ZnSO_4_.7H_2_O	Batch	500	50	No	6.8	28	–	250	Soybean flour	698
												Soy protein isolate	338
				Batch	N.A.	4000	No	6.8± 0.1	28	–	800	Soybean flour (TN: 1.6 g/L, SO: 16 g/L)	742
												Soybean flour (TN: 2.4 g/L, SO: 16 g/L)	840
												Soybean flour (TN: 3.2/L, SO: 16 g/L)	906
												Soybean flour (TN: 1.6 g/L, SO: 23 g/L)	660
												Soybean flour (TN: 2.4 g/L, SO: 23 g/L)	670
												Soybean flour (TN: 3.2 g/L, SO: 23 g/L)	796
21	Saudagar and Singhal, 2007a^$^	MTCC 1142	Soybean flour, dextrin, peptone, KH_2_PO_4_	Batch	N.A.	N.A.	No	7.0± 0.2	25	–	200	Glucose	52.5–75
												Sucrose	37–52.5
												Modified starch	87.5–115
												Soybean oil	107.5–139
												Palm oil	60–139
												Glycerol	97.5–111.5
			Soybean flour, dextrin, rice bran oil, KH_2_PO_4_	Batch	N.A.	N.A.	No	7.0± 0.2	25	–	200	Yeast extract	92.5–112
												Ammonium carbonate	59.5–95
												Ammonium chloride	82.5–96
												Sodium nitrate	17.5–25
												Potassium nitrate	17–20
			Soybean flour, soybean oil, dextrin, yeast extract, K_2_HPO_4_	Batch	N.A.	N.A.	No	7.0± 0.2	25	–	200	Pyruvic acid	475–1025
												α-ketoglutarate	475–480
												L-leucine	475–1175
												L-ornithine	380–580
												L-proline	475–1125
												L-arginine	510–1400
												L-valine	275–500
				Batch	N.A.	N.A.	No	5.0	25	–	200	–	305
								5.5				–	310
								6.0				–	460
								6.5				–	475
								7.0				–	490
								7.5				–	495
								8.0				–	300
								8.5				–	200
22	Saudagar and Singhal, 2007b	ATCC 27064	Glycerol, sucrose, proline, glutamic acid, CaCl_2_.2H_2_O, FeCl_3_, MnCl_2_, NaCl, MgSO_4_.7H_2_O, ZnCl_2_, KH_2_PO_4_	Batch	N.A.	N.A.	N.A.	7.0± 0.2	N.A.	–	N.A.	L-arginine (1–100 mM)	600-1100
												L-proline (1–100 mM)	650-850
												L-ornithine (1–100 mM)	650–750
												L-lysine (1–100 mM)	650-900
												L-leucine (1–100 mM)	30-100
												L-glutamine (1–100 mM)	500-800
												L-threonine (1–100 mM)	750-1700
												L-tryptophan (1–100 mM)	600-620
												L-cysteine (1–100 mM)	420-430
												L-valine (1–100 mM)	20-50
				Batch	N.A.	N.A.	N.A.	7.0± 0.2	N.A.	N.A.	N.A.	KH_2_PO_4_ (1 mM)	724
												KH_2_PO_4_ (10 mM)	878
												KH_2_PO_4_ (100 mM)	779
												KH_2_PO_4_ (200 mM)	524
				Fed-batch	N.A.	N.A.	N.A.	7.0± 0.2	N.A.	N.A.	N.A.	Control	1100
												Glycerol (added every 12 h till 60 h)	1200
												Glycerol (added every 12 h till 72 h)	1280
												Glycerol (added every 12 h till 120 h)	1300
				Fed-batch	N.A.	N.A.	N.A.	7.0± 0.2	N.A.	N.A.	N.A.	Arginine (added every 12 h)	1310
												Threonine (added every 12 h)	1863
23	Efthimiou et al., 2008	ATCC 27064	NH_4_Cl, KH_2_PO_4_, MOPS	Batch	N.A.	50	No	6.8	N.A.	N.A.	N.A.	Glycerol	25
												Sunflower oil	18
												Soybean oil	N.A.
												Flaxseed oil	N.A.
												Rapeseed oil	N.A.
												Olive oil	47
				Batch	2100	1600	0.23 L/min	6.8	30	–	800	Glycerol	55
												Olive oil	45
24	Kim et al., 2009	NRRL 3585	Soya flour, phosphate, MgCl_2_.4H_2_O, FeSO_4_.7H_2_O, ZnSO_4_.7H_2_O	Batch	7000	4500	No	7.0	N.A.	N.A.	N.A.	Olive oil	820
												Palm oil	700
												Corn oil	380
												Triolein	989
												Tripalmitin	410
												Trilinolein	220
25	Salem-Berkhit et al., 2010	ATCC 27064	Starch, soybean flour, phosphate, ZnSO_4_.H_2_O, FeSO_4_.7H_2_O, MnCl_2_.4H_2_O	Batch	500	50	No	7.0	28	–	250	Glycerol	564
												Olive oil	1120
												Cotton seed oil	740
												Corn oil	911
												Castor oil	300
												Coconut oil	380
												Palm oil	580
												Sunflower oil	600
												Linseed oil	700
				Batch	500	50	No	6.0	28	–	250	Glycerol	25
								7.0				Glycerol	564
								8.0				Glycerol	300
								6.0				Olive oil	117
								7.0				Olive oil	1120
								8.0				Olive oil	500
26	Teodoro et al., 2010	ATCC 27061	Glycerol, bacto peptone, malt extract, yeast extract, K_2_HPO_4_, MgCl_2_.4H_2_O, MnCl_2_.4H_2_O, FeSO_4_.7H_2_O, ZnSO_4_.7H_2_O, silicone antifoam, soybean oil	Batch	5000	4000	0.5 vvm	6.8± 0.1	28	–	800	–	509
												Ornithine (0.66 g/L)	560
												Ornithine (0.99 g/L)	480
												Ornithine (1.32 g/L)	380
				Fed-batch	5000	3400	0.5 vvm	6.8± 0.1	28	–	800	–	1390
												Ornithine (3.7 g/L)	1405
												Ornithine (7.4 g/L	1400
												Ornithine (11.1 g/L)	1250
				Fed-batch	5000	3400	0.5 vvm	6.8± 0.1	28	–	800	Feed composition same as media	1050
												Glycerol, ornithine, distilled water only	1080
				Fed-batch	5000	3400	0.5 vvm	6.8± 0.1	28	–	800	Glycerol (120 g/L)	1050
												Glycerol (150 g/L)	1200
												Glycerol (180 g/L)	1506
												Glycerol (240 g/L)	1125
				Fed-batch	10000	8000	0.5 vvm	6.8± 0.1	28	–	800	Ornithine (3.7 g/L)	1560
												Ornithine (5.5 g/L)	1500
27	Cerri and Badino, 2012	N.A.	Glycerol, bactopeptone, K_2_HPO_4_, MgSO_4_.7H_2_O, MnCl_2_.4H_2_O, FeSO_4_.7H_2_O, ZnSO_4_.7H_2_O, MOPS	Batch	N.A.	5000	3 vvm	6.8 ± 0.1	N.A.	N.A.	–	–	454
							4.1 vvm				–	–	269
				Batch	N.A.	4000	No	6.8 ± 0.1	N.A.	N.A.	600	–	269
											800	–	402
28	Costa and Badino, 2012	ATCC 27064	Glycerol, soybean protein isolate, K_2_HPO_4_, MgSO_4_.7H_2_O, MOPS	Batch	500	50	No	6.8	20	–	250	–	1266.2
									25	–		–	631.6
									30	–		–	168.7
				Fed-batch	500	50	No	6.8	20	–	250	Glycerol (1–4 pulses)	1460.0-1534.3
									25				1051.9-1186.6
									30				200.0-440.1
29	Bellão et al., 2013	DSM 41826	Soybean meal, L-lysine, yeast extract, K_2_HPO_4_, MgSO_4_.7H_2_O, CaCl_2_.2H_2_O, NaCl, FeSO_4_.7H_2_O, MnCl_2_.4H_2_O, ZnSO_4_.7H_2_O	Batch	5000	4000	No	6.8 ± 0.1	28	–	800	Glycerol	348.5
												Starch	125.2
				Fed-batch	5000	4000	No	6.8 ± 0.1	28	–	–	Glycerol	982.1
												Starch	469.0

* The study by Romero et al. (1986) expressed maximum clavulanic acid production in a different unit, μg of clavulanic acid per mg of biomass dry weight.

# Values estimated from original studies.

$ For media optimization, studies used factional factorial design matrix (Wang et al., 2005) and L_25_ orthogonal array (Saudagar and Singhal, 2007a).

## Results

### Literature search

**Figure 2:**
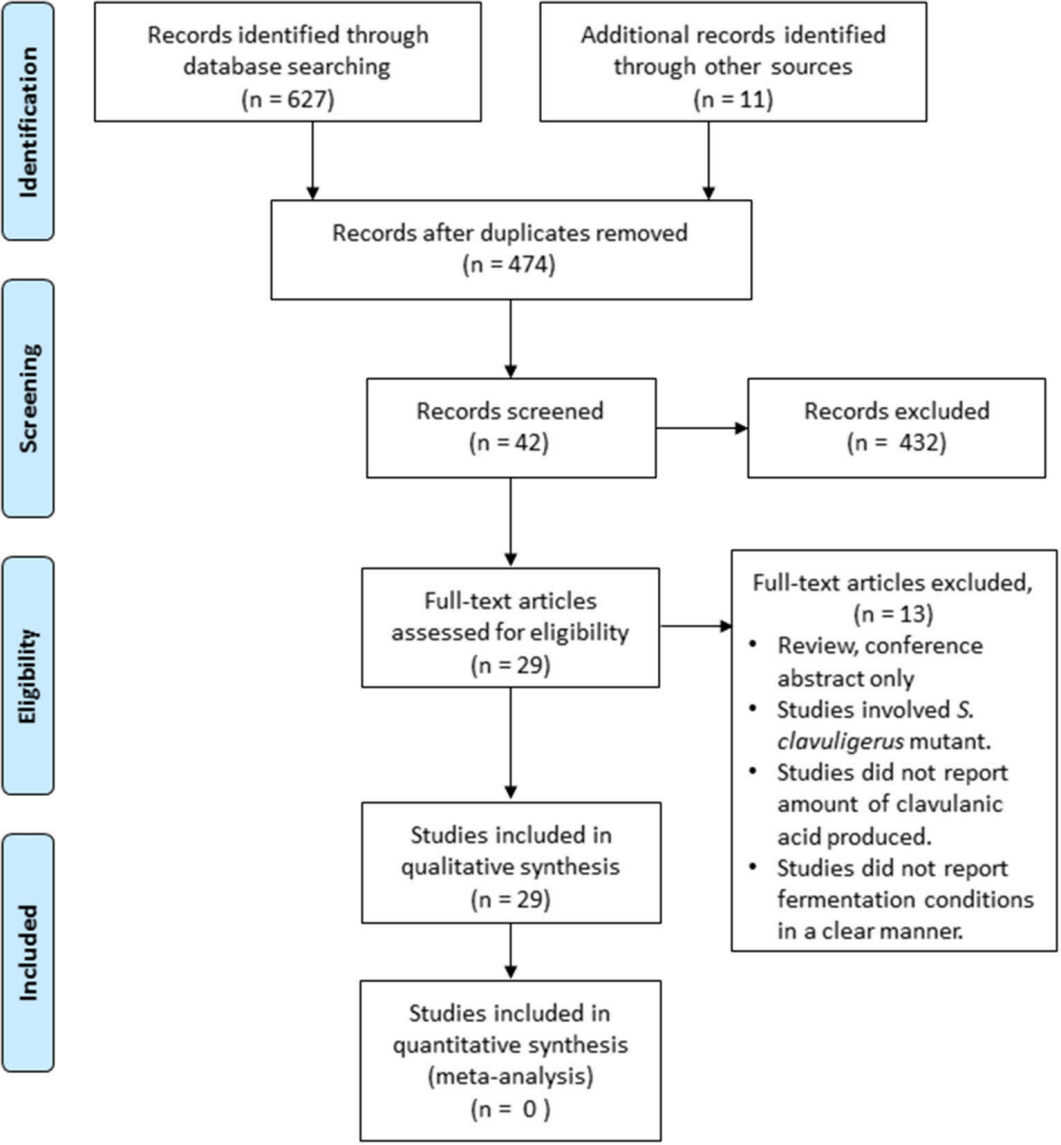
**The PRISMA flow diagram showing the selection process of the studies included in the review**.

The search yielded a total of 627 articles, while an additional 11 articles were obtained from other sources (Figure 2). After the removal of duplicate records, a total of 474 articles were accessed, out of which 432 articles were excluded based on their titles and abstracts. 42 full text articles were reviewed, out of which 29 studies were eligible for the qualitative analysis according to the inclusion criteria (Table 1). The analysis was divided into several categories based on the design of the experiments: (a) utilization of glycerol or starch as sole carbon source, (b) addition of glycerol and different oil in batch fermentation, (c) amino acids as supplements in basal medium, (d) other factors affecting clavulanic acid production in batch fermentation, and (e) comparison between batch and fed-batch fermentations.

#### Utilization of glycerol, starch, or sucrose as sole carbon source

Thakur et al. (1999) and Maranesi et al. (2005) described that different media compositions resulted in different levels of clavulanic acid production. When comparing between glycerol and sucrose as a sole carbon source, Lee and Ho (1996) observed no production of clavulanic acid in media added with glycerol, but higher production of the compound in media with sucrose (3.63 mg/L). Similar findings were also reported by Ives and Bushell (1997) where no production of clavulanic acid was observed in glycerol-containing C-limited media. Meanwhile, another study by Thakur et al. (1999) demonstrated that the addition of dextrin or glycerol as a sole carbon source neither improved nor decreased the production of clavulanic acid. On the contrary, two studies reported a totally different observation—basal media containing glycerol exhibited higher maximum amounts of clavulanic acid as compared to starch (Saudagar and Singhal, 2007a; Bellão et al., 2013). Indeed the maximum amount of clavulanic acid observed in media containing glycerol was found to be 348.5 mg/L, nearly two times higher compared to media with starch as a sole carbon source (Bellão et al., 2013). Additionally, two other studies by Chen et al. (2002) and Saudagar and Singhal (2007a) revealed a biphasic dose response of glycerol; whereby clavulanic acid production was inhibited at concentrations which were either too high or too low.

#### Addition of glycerol and different oil in batch fermentation

Apart from glycerol, several studies (*n* = 6) have also looked at how other oil and unsaturated fatty acids affect the production of clavulanic acid in *S. clavuligerus*. An earlier study revealed a relatively low production of clavulanic acid in media containing different fractions of palm oil or its purified, major constituents (e.g., palmitic acid, stearic acid, lauric acid, oleic acid) (Lee and Ho, 1996). A more recent study in 2009 by Kim et al. (2009) reported maximum clavulanic acid production of 700 mg/mL in media supplemented with palm oil, an intermediate value as compared to other oil sources. The highest clavulanic acid production reported in the same study was observed in media containing triolein (which is a major constituent of palm oil) at 989 mg/L.

Different vegetable oils may stimulate the production of clavulanic acid, as demonstrated by two different studies. In batch fermentation, Efthimiou et al. (2008) described increased clavulanic acid production when olive oil was used in place of glycerol as a sole carbon source; with a maximum concentration of 47 mg/L being recorded, which is nearly double that observed in media containing glycerol (25 mg/L). These results were consistent with another recent study, whereby the addition of olive oil improved clavulanic acid production as compared to glycerol; with a maximum concentration at 1120 and 564 mg/L, respectively (Salem-Berkhit et al., 2010). Relatively high production was also observed in media supplemented with corn oil (911 mg/L), followed by cotton seed oil (740 mg/L), and linseed oil (700 mg/L). Media with castor oil was found to yield the lowest amount of clavulanic acid (300 mg/L). Given that different vegetable oils may have a slight difference in fatty acids and lipid composition, Maranesi et al. (2005) described that when the same concentration of oil was used, the production of clavulanic acid differed slightly between soybean oil, corn oil, and sunflower oil. The greatest concentrations of clavulanic acid using soybean oil, corn oil, and sunflower oil was recorded to be between the ranges of 660–753 mg/L. These results were consistent with a study by Saudagar and Singhal (2007a), as similar concentrations of clavulanic acid was observed in media containing palm oil and soybean oil.

#### Protein and amino acids as supplements in basal medium

The choice of soybean flour or soy protein isolate in fermentation media affects the production of clavulanic acid by *S. clavuligerus* (Gouveia et al., 1999; Wang et al., 2005; Ortiz et al., 2007). The difference in the type of protein as a source of nitrogen was found to affect the production of clavulanic acid as studied by Gouveia et al. (2001). Increased production of clavulanic acid was reported in media containing soybean flour (698 mg/L) rather than soy protein isolate (338 mg/L). Further investigation with soybean flour revealed that different amounts of nitrogen with varied amounts of soybean oil, produced different concentrations of clavulanic acid (660 mg/L – 906 mg/L). Similar findings were reported by Teodoro et al. (2006) as different clavulanic acid concentrations were observed (135–380 mg/L) in media containing varied concentrations of soy protein isolate.

The investigation on the role of amino acids as a source of nitrogen in the production of clavulanic acid began in 1986 (Romero et al., 1986). Several studies focusing on the effects of the amino acids, arginine and ornithine; toward the production of clavulanic acid showed inconsistent results. Romero et al. (1986) reported maximum concentrations of clavulanic acid in a slightly different manner, where 16 μg/dry weight of biomass was observed with arginine; while 9 μg/dry weight of biomass was observed with ornithine. Differing results were recorded by Chen et al. (2003) as media supplemented with ornithine contained higher concentrations of clavulanic acid at 200 mg/L, compared with arginine at 100 mg/L. Nevertheless, recent studies supported the results of Romero et al. (1986); where higher amounts of clavulanic acid were seen in media supplemented with different concentrations of arginine compared to ornithine (Saudagar and Singhal, 2007a,b). One of the studies also tested the effect of other amino acids such as L-proline, L-lysine, L-leucine, L-glutamine, L-threonine, L-tryptophan, L-cysteine, and L-valine (Saudagar and Singhal, 2007b). Among these amino acids, the highest concentration of clavulanic acid was observed in media supplemented with L-threonine; which was not reported in other literature included in this systematic review. Lynch and Yang (2004) tested the influence of L-lysine further by adding degraded clavulanic acid into the fermentation broth. The study suggested that L-lysine is one of the most important amino acids for the production of clavulanic acid, given that fermentation broths with low concentrations of L-lysine (1 g/L) failed to yield any clavulanic acid. Moreover, the addition of degraded clavulanic acid showed improved clavulanic acid production (maximum clavulanic acid concentration at 42 mg/L) as compared to fermentation broths with L-lysine alone (20 g/L, clavulanic acid concentration at 27 mg/L).

#### Other factors affecting clavulanic acid production in batch fermentation

Aside from sole carbon or nitrogen sources, other factors that may affect the production of clavulanic acid include the addition of phosphate, pH, temperature, and agitation or shaking speed. The potential repressive effect of phosphate on clavulanic acid production in *S. clavuligerus* was demonstrated by two selected studies (Lebrihi et al., 1987; Bushell et al., 2006; Saudagar and Singhal, 2007b). The study by Lebrihi et al. (1987) tested two levels of phosphate, 2 and 75 mM. Based on HPLC measurements, the lower concentration of phosphate (2 mM) in fermentation media was found to contain significantly higher levels of clavulanic acid; with maximum concentration observed at 90 mg/L, as compared to 3 mg/L observed in fermentation media containing high concentrations of phosphate (75 mM). Without changing the pH of the media, Saudagar and Singhal (2007b) demonstrated that the addition of phosphate in the production medium showed a biphasic response. At the highest tested concentration of KH_2_PO_4_ (200 mM), the maximum concentration of clavulanic acid dropped drastically to 524 mg/L. Thus the optimum concentration of KH_2_PO_4_ for clavulanic acid production was determined to be 10 mM (with a maximum clavulanic acid concentration recorded at 878 mg/L).

Furthermore, the pH of fermentation media was described as having a profound effect on clavulanic acid yield (Saudagar and Singhal, 2007a; Salem-Berkhit et al., 2010). By using fermentation media with different pH, different levels of clavulanic acid were seen; with maximum concentrations reported at pH 7. Similar patterns of clavulanic acid production were seen in the tested fermentation media regardless of the sole carbon sources used (i.e., glycerol or olive oil). In addition, Costa and Badino (2012) reported that the temperature at which fermentation was carried out may lead to variation in clavulanic acid production. Low fermentation temperature (20°C) resulted in maximum clavulanic acid concentration as high as 1266.2 mg/L as compared to 631.6 mg/L at 25°C and 168.7 mg/L at 30°C.

Aeration or agitation speed throughout cultivation and production was described to affect clavulanic acid yield as well. In fact, the effect of aeration on the production of clavulanic acid can be tested with a direct experiment involving the use of the Erlenmeyer flask with different baffle heights (Lin et al., 2005). The results showed that the flask with a higher baffle height had a slight increase in the production of clavulanic acid (180 mg/L), as compared to a normal Erlenmeyer flask (90 mg/L). Another study showed that agitation speed has a positive correlation with clavulanic acid production (Rosa et al., 2005). At 800 rpm, two flasks with different oxygen flow rates showed similar levels of maximum clavulanic acid concentration, with 475 mg/L obtained from the flask with the lowoxygen flow rate and 482 mg/L from the flask with the low oxygen flow rate. A study by Cerri and Badino (2012) also supported the view that an increase in agitation speed leads to higher production of clavulanic acid, but not oxygen flow. When the oxygen flow was increased to 4.1 vvm, the maximum concentration of clavulanic acid was observed to be 269 mg/L, which is approximately half of the maximum concentration observed with an oxygen flow of 3 vvm (454 mg/L). However, these results were contradictory with a previous study by Belmar-Beiny and Thomas (1991) which showed that there is no significant difference in clavulanic acid production as a result of different stirring speeds, even with same oxygen flow rate.

Besides that, the presence of redox-cycling agents in the production media may influence the production of clavulanic acid (Kwon and Kim, 1998). Five redox-cycling agents were tested—methyl viologen, menadione, plubmagin, phenazine methosulfate, and hydrogen peroxide (H_2_O_2_). All of the redox-cycling agents promoted the production of clavulanic acid, except methyl viologen (9.96–25.89 mg/L). The highest maximum clavulanic acid concentration was described with phenazine methosulfate (63.73–103.56 mg/L), followed by plumbagin (55.76–79.66 mg/L), and H_2_O_2_ (51.78–69.71 mg/L).

#### Comparison between batch and fed-batch fermentations

Among the selected studies, there were a total of seven fed-batch fermentation experiments, with the majority looking at the effect of adding glycerol into the fermenter over a period of time. Most of the studies took similar approaches to study the effect of glycerol in fed-batch fermentations: (a) by maintaining glycerol at a certain level throughout the fermentation period and/or (b) by adding fixed amounts of glycerol at fixed time points (Chen et al., 2002; Neto et al., 2005; Saudagar and Singhal, 2007b; Teodoro et al., 2010; Costa and Badino, 2012). Regardless of the methods, the study showed higher levels of maximum clavulanic acid concentration than control in the fed-batch fermentation. Comparing batch and fed-batch fermentation, fed-batch fermentation systems seemed to generate a higher yield of clavulanic acid (Chen et al., 2002; Neto et al., 2005; Bellão et al., 2013). A study by Bellão et al. (2013) observed a higher maximum clavulanic acid concentration in the latter method at 982.1 mg/L, as compared to 348.5 mg/L. Apart from the addition of glycerol, lower fermentation temperatures also resulted in a higher yield of clavulanic acid, which was also observed in both batch and fed-batch fermentation.

Besides that, the effects of amino acid was also studied using the fed-batch fermentation approach. Following batch fermentation that revealed increased clavulanic acid production by arginine and threonine, Saudagar and Singhal (2007b) showed that by using fed-batch fermentation technologies; the production of the compound could be further increased. Nevertheless, another study reported lower clavulanic acid yield in fed-batch fermentation with the addition of ornithine as compared to batch fermentation (Chen et al., 2003). On top of that, an increase in the amount of clavulanic acid produced was seen in other fed-batch experiments with the addition of glycerol and arginine. Chen et al. (2003) reported that fed-batch fermentation using glycerol, ornithine, and arginine; yielded different amounts of clavulanic acid, whereby the highest amount was demonstrated with glycerol (300 mg/L), followed by arginine (210 mg/L), and the lowest with ornithine (110 mg/L). The addition of glycerol together with either of the amino acids resulted in intermediate values, where the combination of glycerol and arginine produced 130 mg/L; while glycerol and ornithine resulted in 200 mg/L. Meanwhile, Teodoro et al. (2010) investigated the influence of the presence of ornithine in batch and fed-batch fermentation systems on clavulanic acid production. The study did not find any significant changes in maximum clavulanic acid production due to ornithine, regardless of batch or fed-batch fermentation systems. Interestingly, the same study also revealed an insignificant difference in maximum clavulanic acid concentration when the feeding media (which possesses the same composition as the production media) was replaced with distilled water containing only glycerol and ornithine (at same concentrations as the production media).

## Discussion

The microbial fermentation system is important for the discovery and development of pharmaceutical drugs. Clavulanic acid as a β-lactamase inhibitor was initially isolated from *S. clavuligerus* ATCC 27064 using the traditional fermentation system (Higgens and Kastner, 1971; Brown et al., 1976). β-lactamase inhibitors help to prevent drug resistance against β-lactam antibiotics, and allows successful eradication of harmful pathogens. Considering its therapeutic value against infectious diseases, the biosynthesis pathways of clavulanic acid in *S. clavuligerus* have been studied extensively over the years; beginning around 1980s by a research group led by Romero et al. (1986) (Figure 1). The two precursors involved in clavulanic acid biosynthesis, arginine and glyceraldehyde-3-phosphate; undergo a series of enzymatic processes to form the β-lactam inhibitor. Given that both arginine and glyceraldehyde-3-phosphate play an important role in primary metabolism, the production of clavulanic acid could be improved by refining the composition of the fermentation media (Kirk et al., 2000). It is also presumed that *S. clavuligerus* produces higher amount of clavulanic acid when there is adequate supply of these precursors. The wild type strain of *S. clavuligerus* is unable to metabolize glucose, and further molecular studies revealed that the strain lacks the expression of the glucose permease gene (Garcia-Dominguez et al., 1989; Pérez-Redondo et al., 2010).

**Figure 3:**
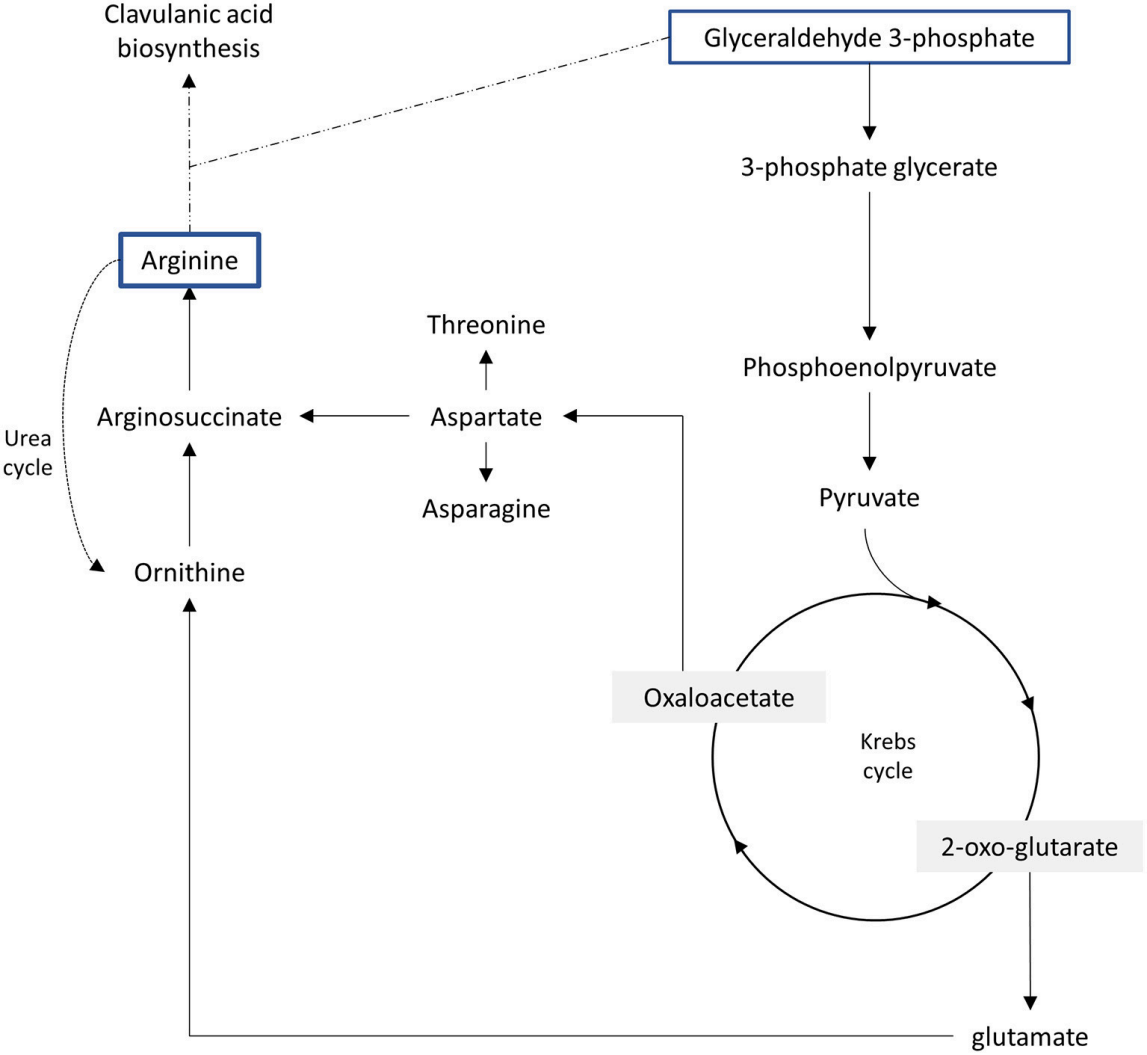
**Important pathways that could affect metabolic pool of clavulanic acid precursors - arginine and glutaraldehyde-3-phosphate (Kirk et al., 2000; Bushell et al., 2006)**.

Based on literature obtained in this study, glycerol was found to be the most popular choice of carbon source in clavulanic acid production; in order to ensure an efficient supply of glyceraldehyde-3-phosphate Once glycerol is metabolized into glyceraldehyde-3-phosphate, it can either enter the clavulanic acid biosynthesis pathway, or be involved in glycolytic or gluconeogenesis reactions (Figure 3). The inclusion of glycerol enhances the production of clavulanic acid as compared to carbohydrates, as glycerol provides a higher energy content on a weight-by weight basis (Efthimiou et al., 2008). As glycerol serves as a backbone for triglycerides, its utilization by *S. clavuligerus* has prompted researchers to study the potential of other oils to be used as a source of carbon. Vegetable oils such as olive oil and corn oil may serve as cost-effective options as they are readily available at a lower cost compared to carbohydrate substrates. As compared to glycerol, vegetable oils seem to be a more attractive source of energy; as a typical oil contains about 2.4 times more energy than glycerol (Stowell, 1987). Other than preventing carbon catabolite regulation, these oils could act as an antifoam, preventing the formation of foam in the media that may impede gas exchange and negatively affect bacteria growth (Friberg et al., 1989; Görke and Stülke, 2008). Overall, the utilization of vegetable oils might be a better choice for clavulanic acid production in fermentation processes compared to glycerol, as these natural oils may further enrich the media with the presence of various polyunsaturated fatty acids (e.g., oleic acid, linoleic acid) (Park et al., 1994). As the addition of olive oil has been shown to greatly improve clavulanic acid production, further investigations on the effect of vegetable oils could suggest the potential of these compounds to be exploited as a cheaper alternative source of carbon in fermentation processes.

On the other hand, many studies have shown the potential of arginine and ornithine as specific precursors for clavulanic acid. These two amino acids are believed to be interconvertible by the urea cycle via the enzymatic action of arginase (Figure 3). Even though presence of the urea cycle in prokaryotes was once considered unusual, several recent studies have reported arginase activity in *S. clavuligerus* (Mendz and Hazell, 1996; Bushell et al., 2006). These reports then further suggest an important role of this pathway in clavulanic acid biosynthesis. Thus, the addition of arginine or ornithine in fermentation media would increase the flow of C5 precursors into the biosynthesis pathway, which then subsequently increase the production of clavulanic acid (Romero et al., 1986; Lynch and Yang, 2004). From the included studies, one study showed that L-threonine improved the production of clavulanic acid (Saudagar and Singhal, 2007b). Indeed the supplementation of L-threonine was found to prevent the anaplerotic flux on pyruvate to synthesize amino acids such as isoleucine, which in turn increased the availability of C3 precursors and eventually enhanced clavulanic acid production (Ives and Bushell, 1997; Bushell et al., 2006; Saudagar and Singhal, 2007b). Even though the study by Saudagar and Singhal (2007b) has emphasized the role of L-threonine in clavulanic acid production, arginine and ornithine have demonstrated relatively strong influence on clavulanic acid production in *S. clavuligerus*. Thus, further studies on L-threonine could eventually shed some light on the importance of this amino acid in clavulanic acid production, particularly using the metabolic flux analysis approach.

Apart from media composition, environmental stress is known to affect the production of secondary metabolites in microorganisms including members of *Streptomyces* genus. The availability of oxygen determines the growth and survival of the bacteria as well as production of secondary metabolites, which includes antibiotics in *S. clavuligerus* (Yegneswaran et al., 1991). Out of the selected studies, there were three studies which highlighted the importance of oxygen control and agitation speed. Fermentation in bioreactors is often paired with computers for precise control of reaction conditions. Rosa et al. (2005) showed that the high speed stirring promoted the production of clavulanic acid, which was associated with a lower amount of biomass production. It is believed that the high speed stirring prevents cell clumping and also causes cell shearing (Toma et al., 1991; Rosa et al., 2005). Additionally, Kwon and Kim (1998) demonstrated that the addition of redox-cycling agents increased clavulanic acid production. The presence of reactive oxygen species in the fermentation media leads to an imbalance in redox status, which could trigger stress and damage microbials, or even lead to cell death (Cabiscol et al., 2010). The breakage and/or damage of bacterial cells upon exposure to such stress in turn, encourage the neighboring cells to produce secondary metabolites in an attempt to survive and protect against the challenge (Toma et al., 1991; Joshi et al., 1996; Rosa et al., 2005).

In addition, temperature and pH control are also crucial for the production of clavulanic acid and stability of the compound. *S. clavuligerus* ATCC 27064 was reported to have optimal growth from 26 to 30°C with no growth above 37°C (Higgens and Kastner, 1971), while most of the selected studies reported that fermentation temperature ranged between 20 and 30°C. Costa and Badino (2012) demonstrated a maximum clavulanic acid concentration of 1266.2 mg/L when fermentation was performed at 20°C. The study also mentioned that lower concentrations of clavulanic acid was observed with the increase in fermentation temperature. Even though a low temperature of 20°C was not favorable for the growth of *S. clavuligerus*, high production yield of clavulanic acid was observed. There are two possible explanations that could lead to this observation: (a) the low temperature places “cold” stress upon the organism which in turn promotes the production of secondary metabolites (including clavulanic acid); (b) the low temperature may have lowered degradation rate of clavulanic acid, thus ensuring the stability of the compound (Beales, 2004; Bersanetti et al., 2005; Jerzsele and Nagy, 2009; Santos et al., 2009; Feng et al., 2011). Similarly, pH of the medium could affect the growth of *S. clavuligerus*, as it is described to grow between pH 5.0 and 8.5, however sporulation is not observed from pH 7.0 to 8.5 (Higgens and Kastner, 1971). Hence, the determination of the optimum temperature and pH for the fermentation process is critical as slight changes in these factors could tip off the balance between the growth of the organism (biomass) and the production of secondary metabolite(s). On top of that, clavulanic acid was shown to be more stable at a neutral pH, as the decomposition rate was described to be higher at acidic or alkaline pH (Jerzsele and Nagy, 2009). Taken altogether, the pH of the media and fermentation temperature may play important roles in clavulanic acid production as these factors could eventually lead to degradation of this valuable compound.

In this review, most of the selected papers used a one-factor-at-a-time method in batch fermentation to study the effect of carbon or nitrogen sources on clavulanic production in *S. clavuligerus*. However, two studies incorporated more complicated analyses in their studies to facilitate multifactorial comparisons. For instance, Wang et al. (2005) proposed using statistical methods to optimize the fermentation media for clavulanic acid production by *S. clavuligerus*. By combining factional factorial design and response surface methodology, the study suggested optimal concentration of three of the most important components identified via factional factorial design—soy meal powder (38.102 g/L), FeSO_4_.7H_2_O (0.395 g/L), and ornithine (1.177 g/L). Meanwhile, Saudagar and Singhal (2007a) designed a slightly different fermentation media by undertaking another statistical approach to optimize fermentation media for clavulanic acid production. By using the L_25_ orthogonal array method, the study suggested optimum concentrations of soybean flour (8.8%), soybean oil (1.2%), dextrin (1.0%), yeast extract (1.5%), and KH_2_PO_4_ (0.2%); with an optimal pH of 7.0 ± 0.2. Thus in designing a fermentation media for clavulanic acid production, it is important to ensure that the media can support the proper growth of *S. clavuligerus;* as well as provide factors that could stimulate the production of valuable secondary metabolites. Following media optimization, several studies have also incorporated another strategy to maximize the production of clavulanic acid—by utilizing fed-batch fermentation systems which represent a high throughput platform as compared to traditional batch fermentation methods.

The main difference between batch and fed-batch fermentation systems is that the latter is frequently monitored with the assistance of sophisticated technologies and allows precise control of the entire fermentation process (Longobardi, 1994; Li et al., 2014). As a scale-up production process, the fed-batch fermentation system often allows an increase in productivity with a concomitant decrease in production cost. At the time of writing, the current report is one of the first that reviews and investigates the effects of fermentation conditions affecting the production of clavulanic acid in *S. clavuligerus;* and further compares the batch and fed-batch fermentation systems for clavulanic acid production. Costa and Badino (2012) and Bellão et al. (2013) reported the utilization of glycerol in fed-batch fermentation systems resulted in a surge in the production of clavulanic acid (observed as a 1.2–2.8-fold increase in maximum clavulanic acid amount, depending on other factors such as temperature). Compared to glycerol, the addition of amino acid (as a source of nitrogen) in fed-batch fermentation showed less of an effect on the production of clavulanic acid. Only three studies reported the effect of amino acid in fed-batch fermentation (Chen et al., 2003; Saudagar and Singhal, 2007b; Teodoro et al., 2010); where one of the studies discovered that the addition of arginine and ornithine did not affect the production of clavulanic acid in both batch and fed-batch fermentation systems (Saudagar and Singhal, 2007b). Chen et al. (2003) discovered that ornithine increased the production of clavulanic acid in batch but not fed-batch fermentation, while arginine increased the production in fed-batch but not batch fermentation. Given that the fed-batch fermentation system entails a scale-up process, researchers would expect a higher yield of end-products (Modak et al., 1986; Thiry and Cingolani, 2002; Hewitt and Nienow, 2007). However, it has been suggested that sometimes a large-scale fed-batch fermentation may not generate similar results as observed in small-scale batch fermentation, as the fed-batch fermentation system involves a more dynamic environment (Hewitt and Nienow, 2007). Nevertheless, fed-batch fermentation appears to be a better fermentation strategy for clavulanic acid production as demonstrated in the selected studies. Likewise, further investigations using this fermentation method could upscale clavulanic acid production and ensure a better understanding of the biosynthesis pathways for this valuable compound.

## Future prospect and conclusion

Over a span of 30 years, the research on the fermentation process for the production of clavulanic acid has gained remarkable interest from the scientific community. In this systematic review, a total of 29 studies was selected after a thorough literature search. It is worth mentioning that there were some inconsistencies in the measurement of clavulanic acid production in *S. clavuligerus* as some studies did not report the standard deviation even though the experiments were carried out in replicate. This then did not allow for results to be synthesized quantitatively and perform a meta-analysis. The majority of the articles highlighted the importance of media composition and supplements in the production of clavulanic acid, particularly glycerol, vegetable oils, and the amino acids, arginine and ornithine. In batch fermentation systems which are commonly used for laboratory-scale production, the utilization of various sugars (e.g., dextrin and sucrose) and glycerol as sole carbon sources in clavulanic acid production requires further investigation; as current studies have reported inconsistent results and the role of these compounds in the biosynthesis pathways is yet to be clearly defined. Further investigation into the role of carbohydrates and glycerol in clavulanic acid biosynthesis would greatly improve the knowledge of media optimization. Nevertheless, the utilization of different oil sources as a sole carbon source and amino acids as a source of nitrogen in the fermentation media seems to have a strong influence on clavulanic acid production in *S. clavuligerus*; followed by other factors such as pH and temperature. Among the vegetable oils, media supplemented with olive oil showed the highest level of clavulanic acid production, which indicates that olive oil contains potentially important nutrients that could improve the production of the antibiotics. Furthermore, amino acids such as arginine and ornithine which could serve as C5 precursors, have also been shown to increase clavulanic acid yield. For the most part, the development of scale-up production tools such as fed-batch fermentation systems could offer a “budget-friendly” method for clavulanic acid production, as this is particularly important for the pharmaceutical industry where production cost is one of the major concerns. With the advancement of next generation sequencing technologies, researchers have identified numerous genes involved in clavulanic acid biosynthesis in *S. clavuligerus* including the clavaminate synthases genes (Medema et al., 2011). By combining this knowledge, further studies involve scale-up productions would be beneficial to identify biosynthetic roles, as well as determine the regulation of these carbon and nitrogen sources in clavulanic acid production in *S. clavuligerus*.

## Author contributions

H-LS and JW-FL contributed to the literature database search, data collection, data extraction, data analysis and writing of the manuscript. H-LS, JW-FL, NC, SAJ, UDP, K-GC, B-HG and L-HL performed data analysis and rationalization of the results. The topic was conceptualized by B-HG and L-HL.

### Conflict of interest statement

The authors declare that the research was conducted in the absence of any commercial or financial relationships that could be construed as a potential conflict of interest.
